# Prescription practices and availability of artemisinin monotherapy in India: where do we stand?

**DOI:** 10.1186/1475-2875-10-360

**Published:** 2011-12-13

**Authors:** Neelima Mishra, Anupkumar R Anvikar, Naman K Shah, Vineet Kumar Kamal, Surya Kant Sharma, Harish Chandra Srivastava, Manoj Kumar Das, Khageswar Pradhan, Hemant Kumar, Yogendra K Gupta, Pooja Gupta, Aditya Prasad Dash, Neena Valecha

**Affiliations:** 1 National Institute of Malaria Research (NIMR), Indian Council of Medical Research, Sector 8, Dwarka, New Delhi 110 077, India; 2 NIMR Field Unit, Rourkela, Orissa, India; 3 NIMR Field Unit, Nadiad, Gujarat, India; 4 NIMR Field Unit, Ranchi, Jharkhand, India; 5 NIMR Field Unit, Guwahati, Assam, India; 6 NIMR Field Unit, Panaji, Goa, India; 7 Department of Pharmacology, All India Institute of Medical Sciences, New Delhi 110 029, India

## Abstract

**Background:**

The World Health Organization has urged all member states to deploy artemisinin-based combination therapy and progressively withdraw oral artemisinin monotherapies from the market due to their high recrudescence rates and to reduce the risk of drug resistance. Prescription practices by physicians and the availability of oral artemisinin monotherapies with pharmacists directly affect the pattern of their use. Thus, treatment practices for malaria, with special reference to artemisinin monotherapy prescription, in selected states of India were evaluated.

**Methods:**

Structured, tested questionnaires were used to conduct convenience surveys of physicians and pharmacists in eleven purposively selected districts across six states in 2008. In addition, exit interviews of patients with a diagnosis of uncomplicated malaria or a prescription for an anti-malarial drug were also performed. Logistic regression was used to determine patient clinical care, and institutional factors associated with artemisinin monotherapy prescription.

**Results:**

Five hundred and eleven physicians from 196 health facilities, 530 pharmacists, and 1, 832 patients were interviewed. Artemisinin monotherapy was available in 72.6% of pharmacies and was prescribed by physicians for uncomplicated malaria in all study states. Exit interviews among patients confirmed the high rate of use of artemisinin monotherapy with 14.8% receiving such a prescription. Case management, i.e. method of diagnosis and overall treatment, varied by state and public or private sector. Treatment in the private sector (OR 8.0, 95%CI: 3.8, 17) was the strongest predictor of artemisinin monotherapy prescription when accounting for other factors. Use of the combination therapy recommended by the national drug policy, artesunate + sulphadoxine-pyrimethamine, was minimal (4.9%), with the exception of one state.

**Conclusions:**

Artemisinin monotherapy use was widespread across India in 2008. The accessible sale of oral artemisinin monotherapy in retail market and an inadequate supply of recommended drugs in the public sector health facilities promoted its prescription. This study resulted in notifications to all state drug controllers in India to withdraw the oral artemisinin formulations from the market. In 2010, artesunate + sulphadoxine-pyrimethamine became the universal first-line treatment for confirmed *Plasmodium falciparum *malaria and was deployed at full scale.

## Background

Artemisinin-based combination therapy (ACT) has been the mainstay treatment for *Plasmodium falciparum *malaria in Southeast Asia for more than 10 years and is now recommended as the first-line treatment throughout the world [1]. Artemisinins produce rapid therapeutic response, reduce gametocyte carriage, and are well tolerated by patients [2]. The World Health Organization (WHO) also recommends the use of artemisinin derivatives only in combination with effective partner drugs to ensure high cure rates and delay resistance. The five formulations of ACT recommended by WHO are artemether + lumefantrine, artesunate + mefloquine, artesunate + amodiaquine, artesunate + sulphadoxine-pyrimethamine and dihydroartemisinin + piperaquine [3].

In India, the national treatment policy prior to 2008 recommended chloroquine for *P. falciparum *and *P. vivax *treatment in areas where therapeutic studies had not been conducted or where treatment failure was less than 10%. However, systematic analyses of chloroquine resistance suggested the phenomenon was widespread [2,4], not focal, and ACT was introduced by the National Vector Borne Disease Control Programme (NVBDCP) as the first-line treatment of falciparum malaria in 117 districts which represented more than 90% of the reported *P. falciparum *cases [5]. Presently all formulations of ACT recommended by WHO, except dihydroartemisinin + piperaquine, are registered with the Drug Controller General of India. In the public sector health system only artesunate + sulphadoxine-pyrimethamine blister packs were recommended though the combination was supplied as loose tablets as well.

The success of a new treatment policy depends on the adherence of health providers to the guideline and the compliance of patients with the recommended treatment. Equally, information on current provider and patient practices is needed to inform and improve future treatment policy. Recent reports note changes in pattern of drug prescription and utilization in public and private health facilities following changes in national treatment policy [6,7]. However, inappropriate treatment practices continue in spite of the availability of evidenced-based guidelines [8]. Informal assessments and earlier studies in the Indian public and private sector suggest a substantial burden of incorrect treatments and an irrational use of antibiotics and injectables [9]. Large-scale evaluations of the state of anti-malarial treatment patterns, including the prescription of artemisinin monotherapy, were not available in India. Therefore, the treatment practices of physicians, patient experiences, and anti-malarial drug availability in pharmacies were evaluated in multiple sites across the country.

## Methods

### Study areas

Six states were selected: Assam, Delhi, Goa, Gujarat, Jharkhand, and Orissa. Out of these, Assam, Jharkhand, and Orissa were priority states for *P. falciparum *control representing 55.1% of the reported burden in the country [5]. The other three states represented different transmission settings to provide a more representative national picture. In Goa, most malaria is associated with high-risk labour migration, while Gujarat is a low transmission setting with both major malaria species, and in Delhi local transmission is of *P. vivax *only. In each state, except Delhi, two districts (third level administrative unit of one to three million population) were selected based on the proportion of *P. falciparum *among all malaria (≥50%) in the district from surveillance data and the introduction of ACT by NVBDCP.

### Study design

A cross-section of conveniently selected health facilities in each site were surveyed during August to November 2008. Different levels of health facilities including district hospitals, primary health centres (PHC), subcentres, and private hospitals or clinics were interviewed. All physicians present in the selected health facility were interviewed. Surveys were also conducted amongst all pharmacists around a 5 km radius of selected health facilities. Finally, exit interviews were conducted in all patients leaving the selected health facility that day with either a diagnosis (clinical or laboratory) of malaria or to whom anti-malarial medicines were prescribed. An earlier study in Jharkhand described 17% artemisinin monotherapy (Mishra *et al*., unpublished data) among treatments. With 90% power and an alpha of 0.05, at least 1, 733 patients would be needed to detect a 20% prevalence in such a setting.

### Data collection

Each interview team included one experienced surveyor and a trained supporting staff. Data was collected using pre-structured and pre-tested questionnaires. Physicians were interviewed about their years of experience in providing health services, educational qualifications, use of blood slide or rapid diagnostic kit (RDK) for diagnosis, and the prescription and frequency of use of different anti-malarials. Patients were interviewed about their symptoms, the diagnostic workup received along with its result, and drugs prescribed (if any). In the case of minor children, guardians/parents were interviewed. Paper records of the visit, tests, and prescription were also examined, if available. Pharmacists were interviewed and asked about the availability and sale of all anti-malarials in their stock.

### Data analysis

A single data entry staff recorded results using Microsoft Excel 2003 and each entry was cross-checked by a senior investigator. Range checks were used to detect implausible values. Data analysis was conducted with STATA v10. Questionnaire responses were summated, physician characteristics were compared by public and private sector status, and diagnostic and treatment indicators were compared by state. Logistic regression was used to determine adjusted odds ratios for facility and clinical factors associated with artemisinin monotherapy. All univariate variables were included in the multivariable model except in the case of collinear predictors where the model with the better fit (lower AIC criterion) was retained.

### Ethics

Verbal consent was obtained from all interviewees, data was de-identified, and the Scientific Advisory Committee of the National Institute of Malaria Research approved the study design and questionnaires. The study was a public health program evaluation conducted at the request of NVBDCP and thus IRB exempt.

## Results

Five hundred and eleven physicians and 1, 832 patients were interviewed in 98 private and 98 public sector health facilities (Table 1) along with 530 pharmacists. No physician, patient, or pharmacists declined an interview. Public sector physicians had fewer years of clinical experience on average but a higher proportion of them had post-graduate training in medicine compared to physicians in the private sector. A high proportion of private sector physicians were non-allopathic trained.

**Table 1:** Characteristics of 511 interviewed physicians by sector, India, 2008

	Public		Private		Total	
**Characteristic**	**n**	**%**	**n**	**%**	**n**	**%**

*State*						

Assam	75	24.6	33	16.0	108	21.1

Goa	46	15.1	20	9.7	66	12.9

Gujarat	47	15.4	45	21.8	92	18.0

Delhi	44	14.4	28	13.6	72	14.1

Jharkhand	55	18.0	50	24.3	105	20.6

Orissa	38	12.5	30	14.6	68	13.3

*Facility type*						

District hospital/nursing home	176	58.1	124	60.5	300	58.8

PHC, subcentre/clinic	101	33.3	46	22.4	147	28.8

Other	28	9.2	35	17.1	63	12.4

*Experience (years)*						

< 2	39	12.9	11	5.4	50	9.8

2-10	94	31.0	56	27.3	150	29.5

10-20	94	31.0	66	32.2	160	31.5

> 20	76	25.1	72	35.1	148	29.1

*Education*						

MBBS	131	43.0	74	35.9	205	40.1

Post-graduate	149	48.9	81	39.3	230	45.0

Non-allopathic	25	8.2	51	24.8	76	14.9

Missing: facility type (1), experience (3)

### Case management practices by physicians for uncomplicated malaria

Most physicians (62.2%) reported using both slides and RDKs for diagnosis. A higher proportion of private sector physicians used RDKs exclusively (9.2%) compared to those in the public sector (1.6%). Private sector physicians 'always' prescribed more artemisinins alone, artemisinin plus other anti-malarials, and less artesunate + sulphadoxine-pyrimethamine (18.5%, 7.8%, 10.2% of prescriptions respectively) versus those in the public sector (6.9%, 3.6%, 14.1%) (Table 2). Chloroquine was the most frequently prescribed anti-malarial drug in both sectors. Statewise 'always' prescription of artesunate alone and of artesunate + sulphadoxine-pyrimethamine was highest in Jharkhand and Orissa, and Goa and Orissa respectively (Table 2).

**Table 2:** Prescribing frequency (%) of different anti-malarials by 511 interviewed physicians by sector or state, India, 2008

	Public					Private				
**Treatment**	**Always**	**Often**	**Some**	**Rarely**	**Never**	**Always**	**Often**	**Some**	**Rarely**	**Never**

Chloroquine	63.6	18.0	6.6	4.3	7.2	57.3	7.3	14.1	3.9	17.5

SP	1.6	5.6	13.4	10.8	68.2	2.9	10.7	6.8	11.2	68.5

Mefloquine	1.0	1.6	7.2	9.2	80.7	1.5	1.5	6.8	5.8	84.5

Quinine	7.2	22.6	23.3	11.8	34.8	10.7	16.5	15.5	17.5	39.8

ASP	14.1	11.2	20.0	8.5	45.9	10.2	12.1	10.2	5.8	61.7

As alone	6.9	20.0	23.3	12.5	37.1	18.5	20.9	16.0	12.1	32.5

As+other	3.6	4.9	10.8	7.2	73.1	7.8	3.9	4.9	4.9	78.6

	Assam					Goa				

Chloroquine	65.7	14.8	9.3	2.8	7.4	87.9	6.1	1.5	0.0	3.0

SP	0.9	6.5	15.7	18.5	58.3	3.0	1.5	4.6	9.1	80.3

Mefloquine	0.0	0.0	3.7	7.4	88.9	4.6	3.0	6.1	12.1	72.7

Quinine	10.2	29.6	29.6	17.6	13.0	6.1	1.5	16.7	22.7	51.5

ASP	4.6	12.0	21.3	8.3	53.7	47.0	12.1	12.1	9.1	18.2

As alone	6.5	18.5	27.8	11.1	36.1	9.1	4.6	4.6	3.0	77.3

As+other	4.6	0.9	1.9	0.9	90.7	13.6	0.0	1.5	3.0	78.8

	Gujarat					Delhi				

Chloroquine	79.4	2.2	1.1	1.1	16.3	55.6	34.7	1.4	0.0	8.3

SP	1.1	5.4	6.5	1.1	85.9	2.8	15.3	18.1	12.5	51.4

Mefloquine	0.0	1.1	1.1	1.1	96.7	2.8	2.8	23.6	8.3	62.5

Quinine	5.4	10.9	10.9	8.7	64.1	2.8	16.7	18.1	9.7	52.8

ASP	1.1	2.2	5.4	1.1	90.2	1.4	5.6	20.8	11.1	61.1

As alone	13.0	15.2	22.8	15.2	33.7	8.3	19.4	11.1	18.1	43.1

As+other	14.1	2.2	1.1	1.1	81.5	1.4	4.2	1.4	2.8	90.3

	Jharkhand					Orissa				

Chloroquine	47.6	10.5	15.2	9.5	17.1	29.4	17.7	29.4	10.3	13.2

SP	3.8	12.4	8.6	11.4	63.8	1.5	2.9	10.3	11.8	73.5

Mefloquine	1.0	1.0	8.6	12.4	77.1	0.0	2.9	1.5	5.9	89.7

Quinine	13.3	31.4	16.2	9.5	29.5	11.8	22.1	29.4	19.1	17.7

ASP	6.7	14.3	14.3	10.5	54.3	27.9	25.0	23.5	4.4	19.1

As alone	15.2	30.5	28.6	15.2	10.5	17.7	30.9	17.7	8.8	25.0

As+other	1.9	1.0	1.9	3.8	91.4	7.4	0.0	0.0	0.0	92.7

305 public (1 missing) and 206 private physicians, As - artemisinin, As alone - artemisinin monotherapy, SP - sulphadoxine-pyrimethamine, ASP - artesunate+SP

### Anti-malarial treatments prescribed to patients

Exit interviews results show a wide range of anti-malarial groups and treatments, as well as primaquine and antibiotics, were prescribed to 93.8% of patients (Table 3). There were fifteen different treatment regimens recorded and the majority of patients received non-artemisinin monotherapy for the treatment of suspected or confirmed malaria. Artemisinin monotherapy represented 14.8% of the total prescriptions while recommended ACT represented 4.9% and other artemisinin combinations were 5.6% of the total. Overall, the co-prescription of primaquine was 34% reaching 87% among confirmed *P. vivax *patients in the public sector and 52% in private health facilities. The co-administration of antibiotics, 13.6% overall, was highest with chloroquine (n = 74) followed by patients without anti-malarial prescription (n = 23).

**Table 3:** Anti-malarial groups and treatments prescribed to patients as well as primaquine and antibiotics from exit interviews, India, 2008

Drug group/treatment	n	%	PQ	Abx
Artemisinin monotherapy	271	14.8	6	75

				

Non-artemisinin monotherapy	1, 241	68.0	520	98

Chloroquine	1155		520	74

Primaquine	5			

Quinine	74			24

SP	7			

				

WHO ACT	89	4.9	37	2

ASP blister	30			2

ASP loose	16		5	

ASP+CQ	37		31	

ASP+Q	3		1	

ASP+CQ+Q	3			

				

Non-WHO ACT	102	5.6	8	35

As+CQ	63		7	19

As+Q	37			15

As+CQ+Q	2		1	1

				

Non-artemisinin combination	15	0.8	7	0

CQ+SP	13		7	

CQ+Q	2			

				

No anti-malarial drug	108	5.9	0	23

Missing	6			

	1832		583	233

CQ - chloroquine, SP - sulphadoxine-pyrimethamine, Q - quinine, As - artemisinin derivative, ASP - artesunate+SP, PQ - primaquine, Abx - antibiotic

### Patient experiences of physician case management

**Figure 1:**
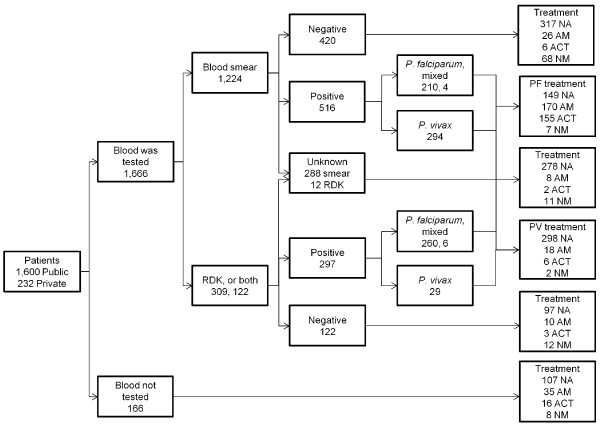
**Flow chart of patient case management from exit interviews, India, 2008**. NA - non-artsesunate treatment, AM - artesunate monotherapy, ACT - WHO or non-WHO ACT, NM - non-antimalarial.

**Figure 2:**
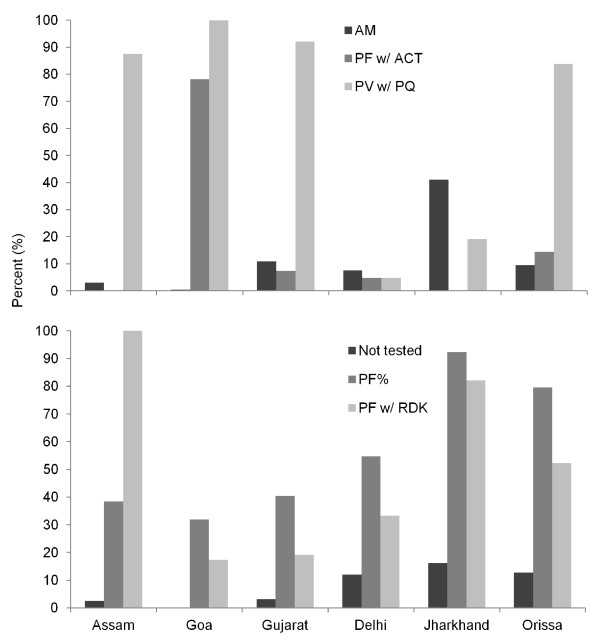
**State wise treatment (top) and diagnosis (bottom) trends from patient interviews, India, 2008**. Treatment legend (top): AM - artesunate monotherapy, PF w/ACT - Diagnosed *P. falciparum *cases prescribed WHO ACT, PV w/PQ - Diagnosed *P. vivax *cases prescribed primaquine. Diagnosis legend (bottom): Not tested - patients who did not receive a blood test, PF% - proportion of *P. falciparum *and mixed infection among diagnosed cases, PF w/RDK - *P. falciparum *cases diagnosed by RDK.

1, 600 patients in public and 232 patients in private health facilities were interviewed. Of these, 91% received some blood test with microscopy as the main diagnostic test (73.5%), followed by RDK (18.5%), and 7.3% were tested with both RDK and microscopy together (Figure 1). The use of artesunate monotherapy was 29.8% among confirmed *P. falciparum *patients, 5.5% for *P. vivax*, 2.7% for those with unknown diagnosis, 6.6% in those with negative reports, and 25.7% in patients with clinical assessment alone (Figure 1). Rational treatment, i.e. a non-anti-malarial prescription, was higher in smear negative patients (16.2%) compared to those who were tested using RDK or both (9.8%). The proportion of clinically diagnosed patients, those receiving artemisinin monotherapy, confirmed *P. falciparum *among total confirmed as well as the proportion of the former diagnosed by RDK were all highest in Jharkhand (Figure 2). Notably, 78% of confirmed *P. falciparum *patients in Goa received ACT (artesunate + sulphadoxine-pyrimethamine). Patients who were prescribed artemisinin monotherapy versus other regimens were compared by facility, patient, and management risks (Table 4). The prescription of artemisinin monotherapy was higher in hospitals, patients 15 years or older, government health facilities, cases screened by RDK, and in *P. falciparum *confirmed patients. Adjusting for multiple risks, patients in Delhi and Jharkhand, exiting private health facilities, 15 years or older, and with mixed or confirmed *P. falciparum *infections had higher odds for receiving an artemisinin monotherapy prescription (Table 4).

**Table 4:** Unadjusted and adjusted site, facility, patient, and management risks for artemisinin monotherapy (n = 273), India, 2008

				Unadjusted		Adjusted	
	**AM**	**Other**	**%**	**OR**	**95%CI**	**OR**	**95%CI**

*State*							

Assam	9	296	3.0	--		--	

Goa	1	217	0.5	0.2	0.0, 1.2	0.0	0.0, 0.4

Gujarat	24	197	10.9	4.0	1.8, 8.9	0.9	0.3, 2.8

Delhi	24	293	7.6	2.7	1.2, 5.9	5.4	1.7, 18

Jharkhand	185	266	41.0	23	11, 49	5.5	2.1, 15

Orissa	30	285	9.5	3.5	1.6, 7.5	0.3	0.1, 1.1

*Facility type*							

Hospital	240	1, 139	17.4	--		--	

Primary clinic	33	415	7.4	0.4	0.3, 0.6	0.6	0.3, 1.1

*Age*							

< 15 yrs	63	444	12.4	--		--	

≥15 yrs	209	1105	15.9	1.3	1.0, 1.8	1.8	1.2, 2.9

*Sex*							

Male	150	957	13.6	--		--	

Female	123	597	17.1	1.3	1.0, 1.7	0.8	0.6, 1.3

*Sector*							

Government	241	1357	15.1	--		--	

Private	32	197	14.0	0.9	0.6, 1.4	8.0	3.8, 17

*Diagnosis*							

Blood Slide	97	1, 122	8.0	--		--	

RDK	119	190	38.5	7.2	5.2, 10	1.5	0.9, 2.7

Both	22	100	18.0	2.5	1.5, 4.2	1.8	0.9, 3.7

None	35	129	21.3	2.9	1.9, 4.4		

*Result*							

Malaria positive	194	621	23.8	--			

Malaria negative	36	503	6.7	0.2	0.2, 0.3		

Unknown	8	298	2.6	0.1	0.0, 0.2		

*Species*							

*P. vivax*	18	306	5.6	--		--	

*P. falciparum*	166	305	35.2	9.3	5.4, 16	4.2	2.2, 7.8

Mixed infection	4	6	40.0	11	2.8, 46	5.0	0.9, 28

Missing: diagnosis type unknown (10), species (10), treatment (5), result and no diagnosis were dropped from adjusted analysis due to colinearity with other variables, AM - artemisinin monotherapy

### Anti-malarial availability and sale in the pharmacy shop

Among 530 pharmacy shops, 72.6% reported the sale of artemisinin derivatives alone in the past year. Of these, 44.2% of sales were injectable derivatives. The most common drug sold across the counter was chloroquine. Goa and Delhi had the high unavailability of artemisinin (46.3%, 55.6%) while the highest proportion of shops having sold injectable artemisinin derivatives were in Assam and Jharkhand (71.3%, 66.1%).

## Discussion

Artemisisin monotherapy was widely available in Indian pharmacies and frequently prescribed by according to both patient and physician interviews. Overall case management varied between the public and private sector as well as region.

### Artemisinin monotherapy

The inappropriate use of anti-malarials in the past has contributed to decreased drug sensitivities today [10]. Recent therapeutic efficacy studies along the Thai-Cambodia border described an increased proportion of patients who were parasitaemic on the third day of follow-up, indicating reduced parasite susceptibility to artemisinins in an area where oral artemisinin monotherapies were widely available [11]. In this study, the easy accessibility, unregulated sale of oral artemisinin monotherapy in the retail market, and inadequate availability of the recommended ACT in public and private health facilities were key contributors. In informal discussions with physicians, other factors hindering the implementation of the new policy were the inertia of practitioners in accepting new treatments, lack of awareness about the new drug policy, and fear of stock outs. The widespread availability of artemisinin monotherapy in both oral and injectable forms, and the different policies surrounding each, also caused prescription confusion. Notably, monotherapy use was strongly associated with private sector care in multivariable analysis where a higher proportion of physicians were non-allopathic. Unsurprisingly, monotherapy use was higher in both *P. falciparum *patients and *P. falciparum *areas. After that however, use was highest among patients who did not receive a blood test and were treated clinically. Dodoo *et al*. reported similar results from Ghana where the prescription of artesunate monotherapy was highest in blood smear positive patients (32.9%) followed by patients without blood tests (21%), underscoring the need for discouraging presumptive treatment [12].

### Rational case management

Proper case management, guided by a laboratory diagnosis, reduces the overconsumption of all anti-malarials, including artemisinin monotherapy. Presumptive treatment in India is discouraged and over 90% of patients in both sectors received a blood test and only a small proportion of patients testing negative or unknown received artemisinin monotherapy. However, many smear or RDK negative patients still received another anti-malarial suggesting a lack of confidence in the diagnosis and/or a lack of concern about the overuse of anti-malarials. In the case of patients tested with RDK alone and microscopy is not available, treatment for vivax malaria may be reasonable as all kits in the public sector were monovalent and detect *P. falciparum *alone. Radical treatment, anti-relapse therapy for *P. vivax *or anti-gametocidal therapy for *P. falciparum*, using primaquine is also an important goal for control. At the time of the study no guidelines for the use of primaquine with ACT in uncomplicated *P. falciparum *malaria were available. For confirmed vivax malaria most patients seen in the public sector received a prescription for primaquine but only half of those in the private sector did. Whether the dose and duration of treatment, as well as counselling for side-effects, were appropriate is unknown. Finally, the co-administration of antibiotics in uncomplicated malaria cases was frequent and is a cause for concern.

### Scaling Up ACT

This study was conducted in 2008 about 1 year after ACT became the recommended treatment of *P. falciparum *cases in limited areas: demonstrated chloroquine resistance, malaria deaths, high proportion of *P. falciparum*. A total of 117 districts were eligible and represented the majority of the reported falciparum malaria burden of the country. ACT blister packs were deployed through NVBDCP for 15 years or older age groups and efforts to produce blister packs for other age groups were ongoing. In this study all of the sampled districts were targeted for ACT except for Delhi. However, the use of ACT for confirmed *P. falciparum *cases was low with the exception of Goa. International attention has focused on identifying sustainable resources for purchasing the needed drugs for widespread deployment through public sector health systems [13]. In India, this is less of a concern where substantial funding was available through the central government. The effective implementation of ACT needs active participation at all levels including members from the periphery health services due to the complex federal structure of the nation and its large size and population. Supply chain improvement and training to dispense the correct dose of ACT is required, particularly to paramedical health workers whose literacy is nominal. The current product profile of ACT used in the country also requires improvement to simplify prescription and promote higher adherence to treatment. They were available exclusively as co-blistered products for adults (> 15 years of age; artesunate in combination with sulphadoxine-pyrimethamine). Lack of availability of different age-specific courses of therapy and short shelf-life (2 years) were other limitations.

### Limitations

This study was conducted in 2008 relatively soon after the first widespread introduction of ACT in country. Even blister packs for different age groups were unavailable necessitating supply of loose tablets of artesunate to be made available in the PHC which may have increased monotherapy use. As the study was conducted for public health needs, important areas were purposively selected rather than random sites and the results may not be population representative. Physician and pharmacist responses may have been biased to appear desirable, however, confidentiality was stressed and participation was 100%.

## Conclusions

Following this study, strict regulation on industry as well as retail outlets was imposed by the Drug Controller General of India leading to a total ban on the sale of oral artemisinin monotherapy since July 2009. Creating awareness among health providers to change clinical practices remains the greatest challenge, particularly in the large private sector. Fixed dose combinations, which prevent the accidental or intentional use of artemisinin monotherapy, are the best available option for future policy. Ensuring access to effective ACT in both public and private sectors, while progressively removing oral artemisinin monotherapies from the market, is essential to provide effective treatment and prevent the development of artemisinin resistance.

## Competing interests

The authors declare that they have no competing interests.

## Authors' contributions

NM, NV were responsible for the design of the project proposal and monitored the study progress. NM, SKS, HCS, KP, MKD, HK and PG carried out the surveys at respective sites. NM VKK, and NKS compiled and analysed the data. NM and ARA wrote the first draft and NV, NKS, APD and YKG corrected the draft and all authors read and approved the final manuscript.

## References

[B1] Ashley EA White NJ Artemisinin-based combinations Curr Opin Infect Dis 2005 6 531 536 10.1097/01.qco.0000186848.46417.6c 16258328

[B2] Valecha N Joshi H Mallick PK Sharma SK Kumar A Tyagi PK Shahi B Das MK Nagpal BN Dash AP Low efficacy of chloroquine: time to switch over to artemisinin-based combination therapy for falciparum malaria in India Acta Trop 2009 111 21 28 10.1016/j.actatropica.2009.01.013 19426658

[B3] World Health Organization Guidelines for the treatment of malariasecond http://www.who.int/malaria/publications/atoz/9789241547925/en/index.html

[B4] Shah NK Dhillon GPS Dash AP Arora U Meshnick SR Valecha N Antimalarial drug resistance of *Plasmodium falciparum *in India: changes over time and space Lancet Infect Dis 2011 11 57 64 10.1016/S1473-3099(10)70214-0 21183147 PMC3068018

[B5] National Vector Borne Disease Control Programme http://nvbdcp.gov.in/

[B6] Kachur SP Black C Abdulla S Goodman C Putting the genie back in the bottle? availability and presentation of oral artemisinin compounds at retail pharmacies in urban Dar- es- Salaam Malar J 2006 5 25 10.1186/1475-2875-5-25 16569252 PMC1444918

[B7] Meremikwu M Okomo U Nwachukwu C Oyo - Ita A Njoku JE Okebe J Oyo- Ita E Garner P Antimalarial drug prescribing practice in private and public health facilities in south - east Nigeria: a descriptive study Malar J 2007 6 55 10.1186/1475-2875-6-55 17480216 PMC1867820

[B8] Wasunna B Zurovac D Goodman CA Snow RW Why don't health workers prescribe ACT? a qualitative study of factors affecting the prescription of artemether-lumefantrine Malar J 2008 7 29 10.1186/1475-2875-7-29 18252000 PMC2266770

[B9] Bhatnagar T Mishra CP Mishra RN Drug prescription practices: a household study in rural Varanasi Indian J Prev Soc Med 2003 34 33 39

[B10] Elyazar IRF Hay SI Baird JK Malaria distribution, prevalence, drug resistance and control in Indonesia Adv Parasitol 2011 74 41 175 21295677 10.1016/B978-0-12-385897-9.00002-1 PMC3075886

[B11] Dondorp AM Nosten F Yi P Das D Phyo AP Tarning J Lwin KM Ariey F Hanpithakpong W Lee SJ Ringwald P Silamut K Imwong M Chotivanich K Lim P Herdman T An SS Yeung S Singhasivanon P Day NP Lindegardh N Socheat D White NJ Artemisinin resistance in *Plasmodium falciparum *malaria N Engl J Med 2009 361 455 467 10.1056/NEJMoa0808859 19641202 PMC3495232

[B12] Dodoo AN Fogg C Asiimwe A Nartey ET Kodua A Tenkorang O Adjei DO Pattern of drug utilization for treatment of uncomplicated malaria in urban Ghana following national treatment policy change to artemisinin- combination therapy Malar J 2009 8 2 10.1186/1475-2875-8-2 19123926 PMC2647941

[B13] Mutabingwa TK Artemisinin- based combination therapies (ACTs): best hope for malaria treatment but inaccessible to the needy! Acta Trop 2005 95 305 315 10.1016/j.actatropica.2005.06.009 16098946

